# Case Report: Pembrolizumab-associated airway-predominant relapsing polychondritis with longitudinal CD8+ T-cell activation and bronchial stenting

**DOI:** 10.3389/fimmu.2026.1999620

**Published:** 2026-10-22

**Authors:** Can Xia, Jie Chen, Xia Fang, Changwen Deng, Xiaoping Zhu, Shuang Ye, Runci Wang

**Affiliations:** 1 Department of Rheumatology, Renji Hospital, Shanghai Jiao Tong University School of Medicine, Shanghai, China; 2 Department of Respiratory and Critical Care Medicine, Shanghai East Hospital, School of Medicine, Tongji University, Shanghai, China

**Keywords:** bronchial stenting, CD8+ T-cell activation, immune checkpoint inhibitor, immune-related adverse event, relapsing polychondritis

## Abstract

Immune checkpoint inhibitors (ICIs) can trigger autoimmune and autoinflammatory toxicities through disruption of peripheral immune tolerance. Relapsing polychondritis (RP) after programmed cell death protein 1 (PD-1) blockade is rare and are described mainly in individual reports. We report a 66-year-old man who developed severe airway-predominant RP after 10 cycles of pembrolizumab for lung adenocarcinoma. Chest CT showed new diffuse tracheal and bilateral bronchial wall thickening and calcification, with severe obstructive ventilatory impairment and systemic inflammation. Exploratory serial peripheral-blood immunophenotyping demonstrated increased proportions of CD38+ and HLA-DR+ cells among circulating CD8+ T cells early in the disease course. Serial measurements showed lower activated CD8+ T-cell proportions during immunosuppressive treatment, in parallel with partial stabilization of respiratory symptoms, pulmonary function and imaging. The patient received sequential glucocorticoids, tofacitinib, methotrexate, abatacept and tocilizumab. Despite partial inflammatory control, subsequent pulmonary infection with severe coughing was followed by pneumomediastinum and progressive structural airway compromise requiring bilateral main-bronchial stenting. One year after stenting, the stents remained *in situ* with no obvious luminal stenosis and pulmonary function remained stable. This case extends the reported spectrum of pembrolizumab-associated RP by documenting an airway-predominant, treatment refractory course with serial immune phenotyping and long-term post-stenting follow-up.

## 1 Introduction

Immune checkpoint inhibitors targeting programmed cell death protein 1 (PD-1) or its ligand can restore antitumor T-cell activity, but can also precipitate immune-related adverse events (irAEs) (1). Rheumatic irAEs encompass diverse inflammatory and autoimmune phenotypes in approximately 10% of ICI-treated patients (2), with inflammatory arthritis among the most frequent phenotypes, yet relapsing polychondritis (RP) remains exceptionally uncommon that no population-based incidence has been established and the literature remains limited to sporadic reports. RP is characterized by inflammation of cartilage-rich structures; tracheobronchial involvement is clinically important because recurrent inflammation may progress to airway-wall destruction, malacia or fixed stenosis (3) This rarity, together with the potential for life-threatening airway involvement, makes recognition of the phenotype clinically important.

The immunopathogenesis of ICI-associated RP is incompletely understood. In a reported case of nivolumab-associated tracheobronchial chondritis, bronchial tissue contained prominent CD3+ T-cell and CD68+ histiocyte infiltration, with comparable CD4+ and CD8+ T-cell infiltration and very few CD20+ B cells (4). Another case described pembrolizumab-associated RP with bilateral auricular involvement, histological cartilage destruction, improvement after pembrolizumab withdrawal, and recurrence after rechallenging (5). A third case described nivolumab-related tracheobronchial chondritis supported by airway imaging and anti-type II collagen antibodies (6). These findings are compatible with a T-cell-predominant inflammatory process, but published cases have largely provided immune observations at a single diagnostic time point. Longitudinal cellular immune monitoring during treatment has rarely been described (7).

Here, we describe a clinically diagnosed pembrolizumab-associated airway-predominant RP phenotype with severe obstructive ventilatory dysfunction and longitudinal peripheral CD8+ T-cell profiling, sequential steroid-sparing therapy, and subsequent structural airway compromise requiring bilateral main-bronchial stenting. The clinical course allowed us to relate serial immune phenotyping to changes in pulmonary function, imaging, corticosteroid exposure and targeted immunosuppression. The later requirement for bilateral bronchial stenting further illustrates that reduction of inflammatory activity does not necessarily reverse established structural airway injury.

## 2 Case description

A 66-year-old man with no relevant previous medical conditions was diagnosed with poorly differentiated lung adenocarcinoma. He received two cycles of carboplatin, pemetrexed and pembrolizumab before tumor resection and mediastinal lymph-node dissection. Postoperatively, he received two cycles of pembrolizumab with cisplatin-based doublet chemotherapy followed by six cycles of pembrolizumab monotherapy, for 10 pembrolizumab cycles in total. He subsequently developed bilateral costal-arch pain and progressive exertional wheeze, and pembrolizumab was suspended.

Compared with chest CT before immunotherapy, CT at symptom onset showed new diffuse thickening and calcification of the tracheal and bilateral bronchial walls (**Figures 1A a, b**). There was no fever, auricular inflammation, saddle-nose deformity or peripheral synovitis. C-reactive protein was 78.55 mg/L and leucocytes were 12.97 × 10^9/L; ESR was normal. ANA was positive at 1:80, whereas RF, anti-CCP antibodies and ANCA were negative. Pulmonary function showed severe obstruction: FEV1 41% predicted, FVC 89.9% predicted and DLCO 83.8% predicted.

**Figure 1:**
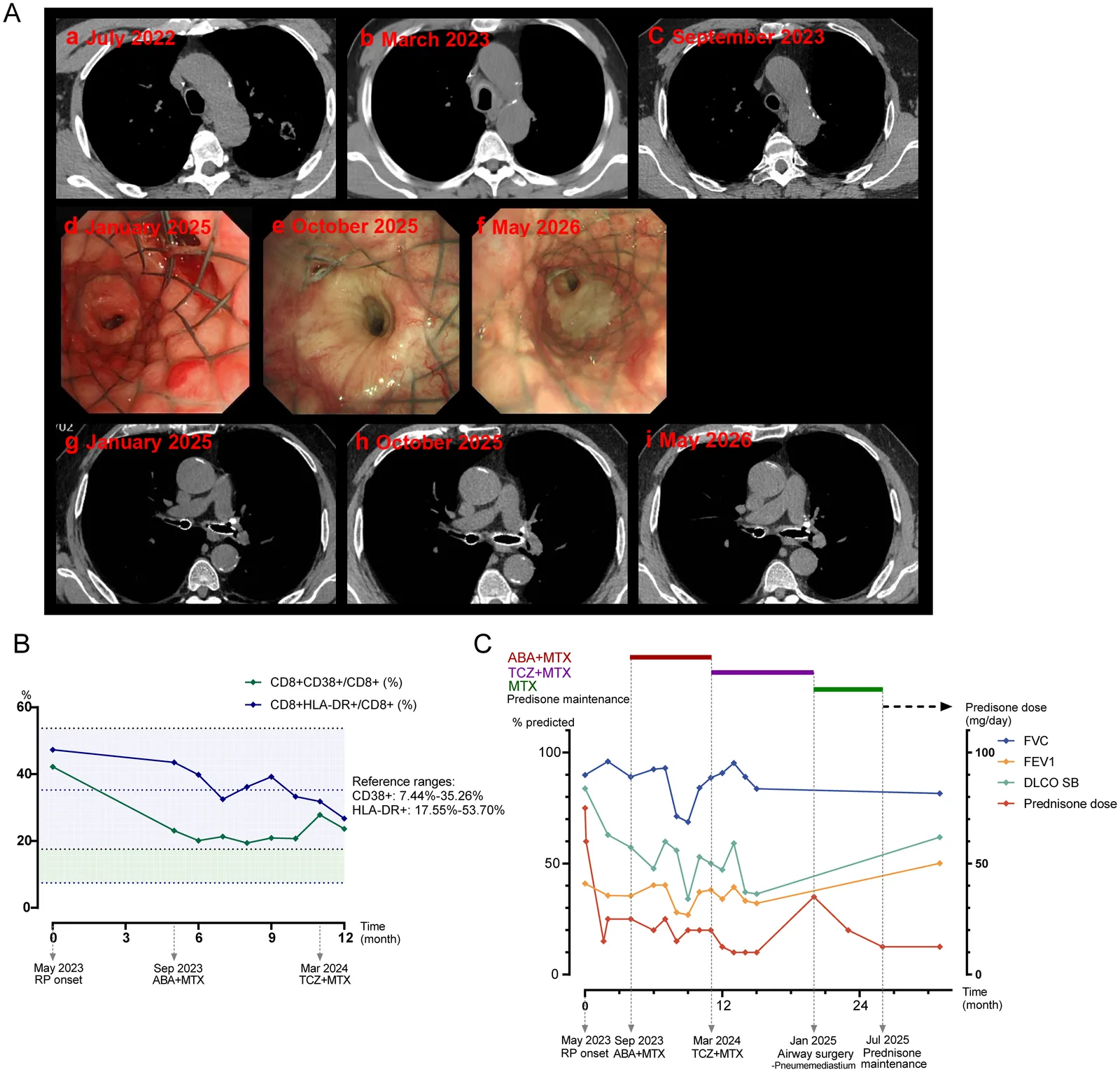
Imaging findings, peripheral CD8+ T-cell activation, and longitudinal clinical course. **(A)** Representative chest CT and bronchoscopy images at different stages of the disease course: **(a)** chest CT before immunotherapy; **(b)** chest CT after 10 cycles of pembrolizumab; **(c)** chest CT after treatment with glucocorticoids and immunosuppressive agents; **(d)** intraoperative bronchoscopy during stent placement at the stenotic airway segment; **(e)** bronchoscopy at 6 months after stent placement; **(f)** bronchoscopy at 16 months after stent placement; **(g)** chest CT after stent placement; **(h)** chest CT at 6 months after stent placement; **(i)** chest CT at 1 year after stent placement. **(B)** Serial proportions of CD38+ and HLA-DR+ cells among circulating CD8+ T cells during treatment. **(C)** Serial changes in pulmonary function, including FVC, FEV1 and DLCO, together with prednisone dose and steroid-sparing treatment during the clinical course. ABA, abatacept; MTX, methotrexate; TCZ, tocilizumab; FVC, forced vital capacity; FEV1, forced expiratory volume in 1 second; DLCO, diffusing capacity of the lung for carbon monoxide.

The temporal relationship with pembrolizumab exposure, new tracheobronchial cartilage abnormalities, inflammatory response, costal symptoms and absence of these airway abnormalities before immunotherapy supported a clinical diagnosis of pembrolizumab-associated tracheobronchial chondritis within the RP spectrum. The absence of classical auricular or nasal inflammation made the presentation atypical and increased the need to distinguish immune-mediated airway disease from infection, tumor recurrence and other causes of central-airway obstruction.

## 3 Diagnostic assessment, immune monitoring and therapeutic interventions

Serial sputum culture and bronchial lavage was performed, together with CT findings, there was no obvious signs of infection or tumor recurrence at the time of presentation. Although the patient did not undergo cartilage biopsy to obtain histology confirmation, based on airway and costal symptoms, multimodal assessment of CT, bronchoscopy and pulmonary physiology, a clinical diagnosis of RP was made, and intravenous methylprednisolone 60 mg/day was administered for 3 days, followed by oral prednisone 60 mg/day together with tofacitinib. Tofacitinib was selected as an oral steroid-sparing targeted synthetic DMARD in the setting of severe steroid-responsive airway inflammation in which prolonged high-dose glucocorticoid exposure was undesirable. Within approximately 1 month, wheeze improved and subsequent CT demonstrated reduced airway-wall thickening (**Figures 1A c**).

Peripheral-blood immunophenotyping was performed serially from disease onset in May 2023 through March 2024 for longitudinal within-patient profiling (**Figure 1B**). Increased proportions of CD38+ cells among circulating CD8+ T cells over healthy control was observed, and the highest observed activation-marker proportions were recorded at the onset of the RP phenotype without signs of pulmonary infection, providing supportive evidence of persistent T-cell activation. Symptoms recurred when prednisone was tapered to 15 mg/day; FEV1 fell to 35.4% predicted and DLCO to 57.3% predicted, suggesting corticosteroid-dependent disease despite the steroid-sparing effort of tofacitinib. Tofacitinib was discontinued, prednisone was increased to 25 mg/day, and subcutaneous abatacept 125 mg weekly plus methotrexate 20 mg weekly was initiated. Abatacept was chosen for its reported success in RP (8), and as a mechanistically relevant steroid-sparing agent because CTLA-4-Ig directly suppresses CD28-mediated T-cell co-stimulation, a pathway of particular interest in a checkpoint-inhibitor-associated T-cell inflammatory phenotype.

The frequencies of CD38+ and HLADR+ CD8 T cells deceased until approximately 4 months of abatacept-based therapy. A pulmonary infection required temporary interruption of abatacept, and pulmonary function deteriorated, while CD38 and HLADR level transiently increased, potentially confounded by the infection. Due to lack of further improvement of pulmonary function on the previous regimen, Tocilizumab was subsequently added as a mechanistically distinct steroid-sparing biologic with published experience in refractory RP while prednisone and methotrexate were continued. Respiratory symptoms, pulmonary function and imaging initially stabilized. Serial immunophenotyping showed lower proportions of CD38+ and HLA-DR+ cells among CD8+ T cells during follow-up, while prednisone and methotrexate were reduced to 10 mg/day and 10 mg/week, respectively (**Figures 1B, C**), although overlapping infection, glucocorticoid tapering and sequential therapies prevent attribution of the response to any single agent.

During a subsequent pulmonary infection in January 2025, severe coughing was followed by pneumomediastinum. At this stage, the clinical problem had evolved beyond active inflammation alone to include major structural airway compromise. Bilateral main-bronchial stents were placed to stabilize the central airways (**Figures 1A d, g**). After the procedure, prednisone was gradually tapered to 12.5 mg/day and methotrexate was maintained at 10 mg/week during monthly follow-up.

## 4 Follow-up and outcomes

The procedure achieved successful airway stent deployment and clinical stabilization. Serial bronchoscopy and CT demonstrated both main-bronchial stents *in situ* and persistent stent patency in October 2025 and May 2026. Diffuse thickening and calcification of the tracheal and bilateral bronchial walls persisted, but no obvious luminal stenosis was seen (**Figures 1A d–i**). Pulmonary function remained stable (FEV1 50.1% predicted, FVC 81.6% predicted and DLCO 61.9% predicted **Figure 1C**). PET-CT demonstrated bilateral multifocal costal inflammation without evidence of recurrent solid tumor. The maintained airway patency at 1 year supports a durable mechanical benefit in this individual, while the persistent structural abnormalities emphasize that control of immune inflammation did not fully reverse established airway damage. The complete timeline of clinical presentatino, treatment and follow-up was summarized in **Table 1**.

**Table 1:** Timeline of the clinical course.

Time	Clinical event	Key findings	Management/outcome
July 2022	Baseline cancer assessment	No tracheal or bronchial wall thickening on CT	Systemic anticancer therapy followed by tumor resection
March 2023	Onset of airway symptoms	Bilateral costal-arch pain, exertional wheeze; diffuse tracheal and bilateral bronchial wall thickening/calcification; FEV1 41% predicted	Pembrolizumab stopped; high-dose glucocorticoids and tofacitinib
April 2023	Initial response	Improved wheeze and reduced airway-wall thickening	Prednisone taper initiated
September 2023, during taper to prednisone 15 mg/day	Clinical relapse	FEV1 35.4% predicted; DLCO 57.3%; highest CD38+ and HLA-DR+ proportions among CD8+ T cells during the early disease course	Prednisone increased; tofacitinib stopped; abatacept 125 mg/week + methotrexate 20 mg/week
March 2024	Pulmonary infection	Pulmonary-function deterioration	Abatacept temporarily interrupted; tocilizumab subsequently added with prednisone and methotrexate
January 2025	Pulmonary infection with severe coughing	Pneumomediastinum	Escalation to interventional airway management
	Central airway intervention	Bilateral main-bronchial stent placement	Prednisone subsequently tapered to 12.5 mg/day; methotrexate 10 mg/week maintained
May 2026	Long-term follow-up	Both main-bronchial stents *in situ*; persistent diffuse airway-wall thickening/calcification but no obvious luminal stenosis; FEV1 50.1%, FVC 81.6%, DLCO 61.9%; PET-CT showed bilateral multifocal costal inflammation and no recurrent solid tumor	Continued surveillance; durable airway patency at 1 year

## 5 Discussion

This case provides a detailed longitudinal view of ICI-associated RP. It is recognized that rheumatic irAEs may not fulfil conventional rheumatic-disease classification criteria (2). In this case, the aggregate evidence—new characteristic airway-cartilage abnormalities after pembrolizumab, severe obstructive physiology, inflammatory activity, glucocorticoid responsiveness, later costal inflammation and exclusion of tumor recurrence—supports an airway-predominant RP diagnosis despite the absence of histological confirmation. Contemporary reviews emphasize that tracheobronchial RP is assessed by a multimodal combination of CT, bronchoscopy and pulmonary function, and that bronchoscopy itself can carry procedural risk in patients with severe airway disease (3, 9). Most published reports of ICI-RP have focused on clinical phenotype, imaging and treatment response, with immunological data obtained mainly at diagnosis. In contrast, repeated assessment of CD38 and HLA-DR expression among circulating CD8+ T cells in our patient allowed immune activity to be followed across changes in corticosteroid exposure and steroid-sparing treatment. The findings do not establish a disease-specific biomarker, but they add temporal information that complements the T-cell-predominant bronchial infiltrates previously reported in nivolumab-associated tracheobronchial chondritis (4). A comparison of ICI-RP cases was summarized in **Table 2**.

**Table 2:** Comparison with selected previously reported ICI-associated RP/chondritis cases.

Report	ICI	Predominant site	Diagnostic support	Immune findings	Treatment	Airway outcome
Mutoh et al. (5)	Pembrolizumab	Bilateral auricles	Auricular biopsy; recurrence on pembrolizumab rechallenge	No serial cellular phenotyping reported	ICI withdrawal; no immunosuppressant required initially	No reported airway involvement
Kuba et al. (6)	Nivolumab	Tracheobronchial tree	CT; positive anti-type II collagen antibody	No serial cellular phenotyping reported	Systemic corticosteroids	Clinical/radiological improvement
Someya et al. (4)	Nivolumab	Trachea and bilateral bronchi	CT, FDG-PET/CT, bronchoscopy and bronchial biopsy	T-cell-predominant tissue infiltrate with CD4+ and CD8+ cells	High-dose corticosteroids	Rapid symptomatic and CT improvement
Present case	Pembrolizumab	Airway-predominant; costal inflammation	Serial CT, bronchoscopy and pulmonary function; no airway/cartilage biopsy	Exploratory serial peripheral CD38+/HLA-DR+ CD8+ measurements;	Glucocorticoids, tofacitinib, methotrexate, abatacept, tocilizumab; later stenting	Progressive structural airway disease requiring bilateral main-bronchial stents and long-term follow-up

PD-1 blockade enhances T-cell activation by interrupting an inhibitory pathway that normally restrains effector responses and contributes to peripheral tolerance (1). In this context, the emergence of airway chondritis after pembrolizumab and the accompanying activated CD8+ phenotype is biologically coherent with an ICI-triggered T-cell process. However, several limitations require caution. CD38^hi^ CD8 T cells were expanded in ICI-arthritis over rheumatoid arthritis and psoriatic arthritis (10), but CD38 and HLA-DR are activation markers confounded by infection as well. In this case, the highest within-patient levels were observed at the initial airway-RP presentation before documented pulmonary infection, while subsequent infection was accompanied by modest and transient increase of these markers during transition from abatacept to tocilizumab. We therefore cannot fully separate irAE-related activation from infection-related or treatment-related effects at every time point, but infection does not account for the whole longitudinal pattern. The value of these data lies in providing serial cellular context that is absent from most previously reported ICI-associated RP cases.

The treatment course also illustrates the difficulty of translating an immunological signal into a single mechanistic therapy. The therapeutic sequence was grounded in available RP evidence and a steroid-sparing strategy rather than empirical switching without rationale. Tofacitinib was used initially as an oral steroid-sparing targeted synthetic DMARD during a severe steroid-responsive inflammatory phase. Abatacept reduces T-cell co-stimulation through CTLA-4-Ig, whereas tocilizumab blocks IL-6 receptor signalling. A systematic review of 177 patients with RP reported pooled response rates of approximately 72% for abatacept, 66% for tocilizumab and 56% for methotrexate, although these estimates were derived from uncontrolled heterogeneous reports (8). In our patient, lower activated CD8+ T-cell proportions accompanied partial clinical stabilization during sequential immunosuppression, but co-treatment with glucocorticoids and methotrexate precludes attribution of the effect to abatacept or tocilizumab individually.

A second major insight is the dissociation between immune-inflammatory activity and irreversible airway mechanics. The patient initially showed symptomatic, physiological and radiological improvement during immunosuppression, yet subsequently developed pneumomediastinum and required bilateral main-bronchial stenting. This suggests that once cartilage injury, airway-wall weakness or fixed structural change has developed, further deterioration may occur even when inflammatory activity is partially controlled with discontinuation of ICI and multiple immunosuppressants. The concept that inflammatory control may not reverse established structural airway damage is well recognized in general RP, however, such a transition is particularly relevant in the context of irAE, where delayed recognition of airway-predominant RP can permit structural complications before classical auricular or nasal features appear (3, 11).

Bronchial stenting should therefore be interpreted in this case as treatment of the mechanical consequence of airway damage rather than treatment of the underlying autoimmune process. Previous RP airway series have shown that stenting can improve dyspnea, lung function and exercise tolerance, but long-term complications including mucus retention and granulation tissue are common (12–14). In our patient, absence of obvious luminal stenosis 1 year after stenting supports sustained mechanical benefit, but does not indicate immunological remission and requires continued airway surveillance (15).

The absence of biopsy and external immune controls limits diagnostic and mechanistic certainty, and infection complicates interpretation of some later immune and pulmonary-function measurements. The principal strength of this report is the integration of pretreatment imaging, longitudinal CT and pulmonary-function testing, serial CD8+ activation-marker measurements, treatment exposure and long-term post-stenting follow-up. This multimodal trajectory provides a clearer distinction between immune activity and structural disease than a single diagnostic snapshot.

## 6 Conclusion

Pembrolizumab-associated RP can present as airway-predominant disease without classic auricular or nasal involvement. In this patient, the diagnosis was clinical and supported by multimodal longitudinal evidence despite the absence of histological confirmation. Exploratory longitudinal CD38+ and HLA-DR+ CD8+ T-cell proportions provided a cellular correlate of the clinical course, while later measurements were partly confounded by infection and treatment changes. The later need for bronchial stenting demonstrates that partial control of immune inflammation may not reverse established structural airway injury. Combining serial immune phenotyping with pulmonary physiology, imaging and multidisciplinary airway assessment may improve mechanistic understanding and clinical monitoring, although the biomarker value of circulating activated CD8+ T cells remains unproven.

## 7 Patient perspective

The disease course was complex and repeatedly relapsing, and my family and I initially had little understanding of how immunosuppressive treatment should be used after cancer immunotherapy. With the help of longitudinal immune monitoring and adjustment of immunosuppressive therapy, my pulmonary function improved. During several critical episodes with concurrent infections, immune monitoring allowed immunosuppressive treatment to be adjusted appropriately and helped to control the disease. When severe airway and pulmonary structural damage developed, timely surgical intervention was crucial in managing a life-threatening situation. We hope that sharing this diagnostic and treatment experience will help more clinicians, patients, and families better recognize and understand this rare condition.

## 8 Methods and materials

For flow-cytometric profiling, cells were freshly collected and routinely processed with density centrifugation using Ficoll-Paque Plus (GE Healthcare). Antibody panel included FITC-anti human-CD3 (OKT3), BUV395 anti-human CD4 (RPA-T4), BUV496 anti-human CD8 (RPA-T8), Percp/cy5.5 anti-human CD38 (HIT2), and APC anti-human HLA-DR (L243) antibodies. Cells were incubated with antibody cocktails at 4°C for 20 minutes and washed twice with FACS buffer before acquisition. Cells were passed through a 70-μm filter and acquired on a BD LSRFortessa. Data were analyzed using FlowJo v10.8.1.

## Data Availability

The original contributions presented in the study are included in the article/supplementary material. Further inquiries can be directed to the corresponding author.
